# HnRNP Q Has a Suppressive Role in the Translation of Mouse Cryptochrome1

**DOI:** 10.1371/journal.pone.0159018

**Published:** 2016-07-08

**Authors:** Ilgye Lim, Youngseob Jung, Do-Yeon Kim, Kyong-Tai Kim

**Affiliations:** 1 Department of Life Sciences, Pohang University of Science and Technology (POSTECH), Pohang, Gyeongbuk, Republic of Korea; 2 School of Medicine, CHA University, Seongnam, Republic of Korea; 3 Division of Integrative Biosciences and Biotechnology, Pohang University of Science and Technology (POSTECH), Pohang, Gyeongbuk, Republic of Korea; 4 Department of Pharmacology, School of Dentistry, Brain Science and Engineering Institute, Kyungpook National University, Daegu, Republic of Korea; Karlsruhe Institute of Technology, GERMANY

## Abstract

Precise regulation of gene expression is especially important for circadian timekeeping which is maintained by the proper oscillation of the mRNA and protein of clock genes and clock-controlled genes. As a main component of the core negative arm feedback loops in the circadian clock, the Cry1 gene contributes to the maintenance of behavioral and molecular rhythmicity. Despite the central role of Cry1, the molecular mechanisms regulating expression levels of Cry1 mRNA and protein are not well defined. In particular, the post-transcriptional regulation of Cry1 mRNA fate decisions is unclear. Here, we demonstrate that hnRNP Q binds to mCry1 mRNA via the 5′UTR. Furthermore, hnRNP Q inhibits the translation of mCry1 mRNA, leading to altered rhythmicity in the mCRY1 protein profile.

## Introduction

Most living creatures from cyanobacteria to humans have daily physiological and behavioral rhythms. The formation of these 24-hour rhythms, called "circadian rhythms", is based on the rotation of the earth over a nearly 24-hour period. Although the light-dark cycle resulting from the earth's spin is definitely responsible for synchronizing and entraining the circadian physiologies of living organisms, this circadian rhythm can be maintained in constant darkness for a while, due to the endogenous circadian clock system [1]. In other words, living organisms already have self-sustained, entrainable circadian rhythms, which can be adjusted in response to light stimulation.

The endogenous oscillators are composed of an autoregulatory transcription–translation feedback loop (TTFL) of clock genes [2]. This molecular network of the core clock genes is clearly defined. BMAL1 and CLOCK proteins form a heterodimer which activates the transcription of three Period (Per) genes, two Cryptochrome (Cry) genes, Rev-erb α, and Ror α. This network forms a positive regulatory loop of the circadian clock system [3]. A negative regulatory loop is induced by the formation of the PER:CRY heterodimer [4]. This complex inhibits the action of the BMAL1:CLOCK heterodimer, resulting in interlocked molecular oscillations of core clock genes and clock controlled genes [5–7]. The transcription of Bmal1 can be positively and negatively regulated by ROR α and REV-ERB α, respectively.

Among the core clock genes, Cry genes have been highly focused due to their important role in various organisms. In plants, Cry genes are involved in light-dependent signaling for flowering time [8] and period length control [9]. In drosophila, CRY protein levels are dramatically regulated by light exposure [10]. Since Cry genes are involved in the resetting of circadian rhythms, Cry mutants showed poor synchronization to light-dark cycles or hypersensitive circadian responses to light [11]. Also in mammals, Cry genes had been reported as circadian photoreceptors. Cry1 and Cry2 are expressed in the retina [12], and targeted disruption of either of the two mouse Cryptochrome (mCry) genes results in abnormal light responses [13].

However, the light-independent roles of Cry genes have been focused in the circadian clock in mammals. CRY proteins inhibit the transactivation activity of CLOCK-BMAL1 through direct interaction with CLOCK and BMAL1 [14]. In cultured SCN derived from mCry-null mice, circadian rhythms are dampened in a few cycles [15]. Cry1^−/−^ mice display approximately 1-hour shorter circadian rhythms and Cry double knockout mice (Cry1^−/−^;Cry2^−/−^) show arrhythmic phenotypes. In addition, circadian expression of the clock genes including Per1 and Per2 is also abolished when Cry genes are deficient [16]. Cry genes also have critical roles in several other physiological processes, such as endocrine system, metabolism, and immune responses. For example, mCry-deficient mice showed salt-sensitive hypertension caused by abnormal synthesis of the mineralocorticoid aldosterone [17]. CRY proteins suppress the expression of proinflammatory cytokines through regulating NF-κB and protein kinase A (PKA) signaling [18]. The absence of Cry genes increases the number of activated T cells and the production of TNF-α, IL-1β and IL-6, leading to induction of arthritis [19]. In addition, CRYs modulate Creb activity and hepatic gluconeogenesis [20].

Given the importance of Cry genes in mammalian circadian timekeeping and other physiological processes, the spatio/temporal expression of Crys should be tightly regulated. It has been well described that the CLOCK:BMAL1 complex rhythmically activates the transcription of Cry genes by binding to E-box motifs [21]. In line with this, H3 histone acetylation and the RNA polymerase II binding pattern exhibit circadian rhythmicity in the promoter region of Cry genes [22]. The mechanisms of post-translational activation or degradation of CRY proteins have also been extensively studied. Ubiquitin ligases, including FBXL3 [23, 24] and FBXL21 [25, 26], and the AMPK signaling cascade [27] target CRY proteins for degradation to maintain robust oscillation of CRYs. For development of clock-based therapeutics against several diseases, identification of small molecules that can modulate the activities of CRY proteins is actively under investigation [28]. On the other hand, however, the posttranscriptional regulation of Cry mRNAs has been rarely studied. Recently, it was reported that only 22% of mRNA cycling genes are controlled by circadian *de novo* transcription [29], suggesting that circadian regulation of gene expression at the post-transcriptional level is much more important than previously thought.

Among several RNA-binding proteins, heterogeneous nuclear ribonucleoproteins (hnRNPs) play fundamental cellular roles such as DNA transcription, mRNA splicing, export, degradation, and translation. There are approximately 20 proteins named hnRNPs A-U in hnRNP family, and we previously reported that some of hnRNPs are involved in mammalian circadian clock system. For example, by binding rhythmically to the Rev-erb α 5′UTR, hnRNP Q enhances the translation of Rev-erb α mRNA in a phase-dependent manner and contributes to Rev-erb α protein oscillation [30]. However, until now, suppressive function of hnRNP Q in the translation of clock genes has not been reported. Furthermore, the role of hnRNP Q in mCry1 protein expression was not studied yet. Here, we demonstrate that hnRNP Q specifically interacts with 5′UTR of mCry mRNA and acts as a suppressor in the translation of mCry mRNA.

## Results

### hnRNP Q inhibits the translation of mCry1 mRNA

Given the central position of Cry genes in the mammalian circadian clock, the robust rhythmicity of CRY proteins should be maintained and the oscillation phase of CRYs should be precisely controlled throughout life. Previously, we already showed that transcriptional regulation alone was not sufficient to generate the protein oscillation of clock genes [30]. Fine-tune regulation at post-transcriptional steps is indispensable to maintain circadian rhythm.

RNA-binding proteins are critical factors in determining the fate of mRNAs. RNA-binding proteins are involved in every step of mRNA processing, including RNA splicing, mRNA export, translation, degradation, editing, and polyadenylation. The exact number of RNA-binding proteins is still unknown. Recently, Castello et al. identified 860 proteins within the mRNA interactome [31]. Gerstberger and colleagues reported that over 1,500 proteins can bind to RNAs [32]. Among them, however, hnRNP D is the only factor demonstrated to have post-transcriptional roles on Cry mRNA [33,34].

**Fig 1:**
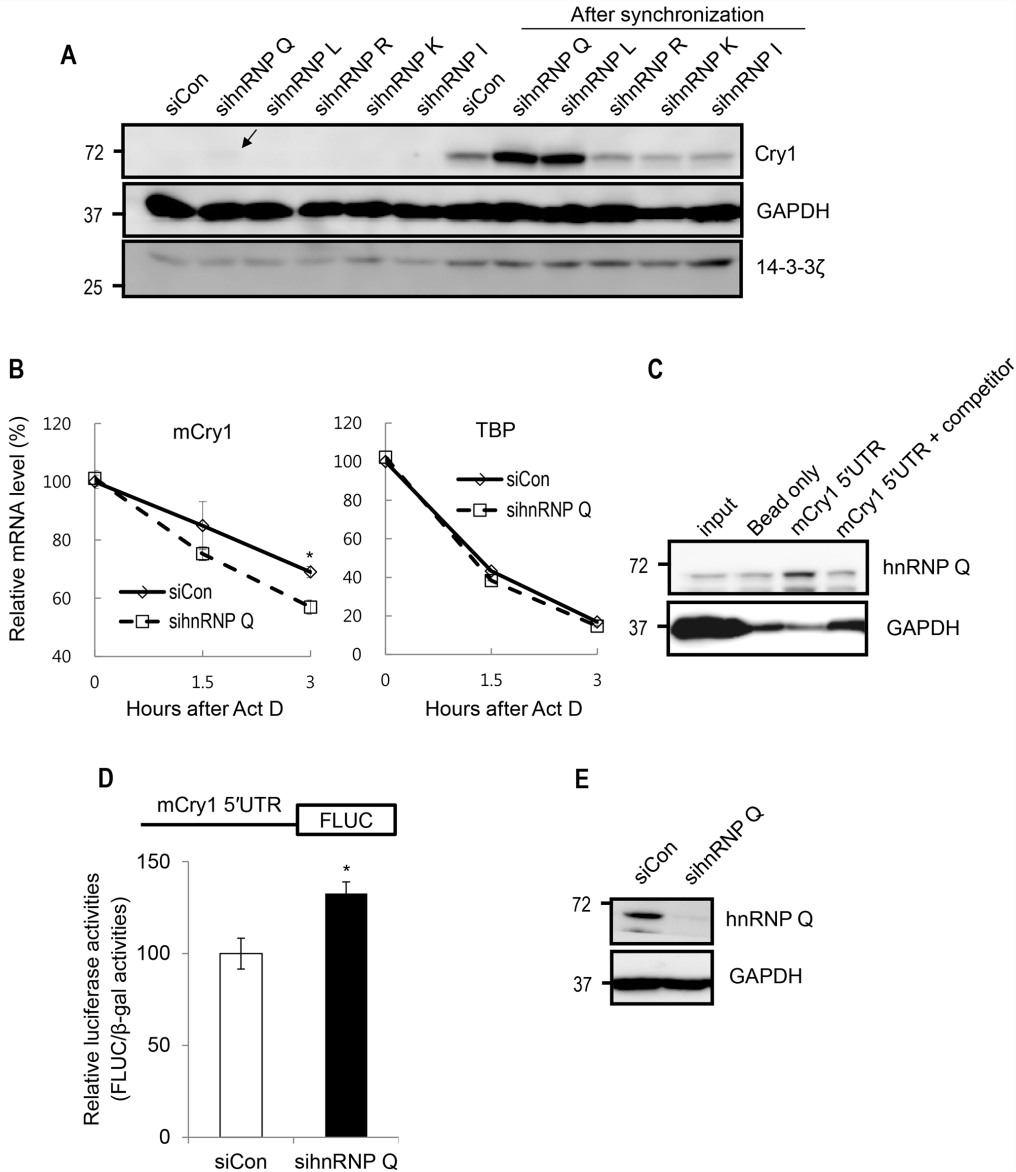
hnRNP Q binds to the 5′UTR of mCry1 and suppresses its translation. (A) RNAi screening in NIH3T3 cells. Five different siRNAs targeting different hnRNPs, including hnRNP Q, were transfected into cells. Control siRNA was used as a negative control. The level of mCRY1 protein was analyzed by Western blotting. On the right side, the circadian phases of cell populations were synchronized by temporal treatment with 100nM dexamethasone, to clearly check the collective effect of hnRNP downregulation on each cell. At 12 hours after synchronization, samples were analyzed. GAPDH and 14-3-3ζ were used as a loading control. The arrow indicates weakly detected mCRY1 protein. (B) mRNA stability of endogenous mCry1 under hnRNP Q downregulation. mRNA degradation kinetics of mTBP was also evaluated as a control. Error bars represent the SEM of three independent experiments. *P<0.05. (C) Identification of the interaction between the mCry1 5′UTR and hnRNP Q by RNA affinity purification followed by immunoblotting. (D) The translation enhancement mediated by the 5′UTR of mCry1 after reduction of hnRNP Q is shown. The 5′UTR of mCry1 was inserted at the upstream of the Fluc coding sequence. Fluc activity was normalized with β-Gal activity. Error bars represent the SEM of seven independent experiments. *P<0.05. (E) Knockdown of hnRNP Q was confirmed by immunoblotting.

Although both CRY1 and CRY2 function as negative transcriptional regulators and slow the clock, CRY1 is more preeminent than CRY2 [35]. In addition, the 5′-untranslated region (UTR) of mCry2 mRNA is not clearly defined. For these reasons, in this study, we focused on the post-transcriptional control of mCry1 mRNA. To identify a new protein that regulates the fate of Cry1 mRNA, we performed RNAi screening with five different siRNAs. Five hnRNPs were downregulated, and the amount of CRY1 protein was analyzed. Among them, silencing hnRNP Q enhanced the CRY1 protein level, compared to the control. The upregulation of the CRY1 protein level was more dramatic when cells were synchronized with dexamethasone (Fig 1A). Although CRY1 protein expression was also affected by knockdown of other hnRNPs (labeled as 1–4 in Fig 1A) and several hnRNPs were predicted to interact with mCry1 UTRs through computational algorithm named RBPmap [36] (S1 Fig), we focused on the role of hnRNP Q which seemed to be the most potent regulator among those tested. To determine whether the upregulated CRY1 protein level was driven by mCry1 mRNA stabilization, we analyzed the mCry1 mRNA decay kinetics under hnRNP Q silencing. Interestingly, hnRNP Q downregulation was rather to promote the degradation of mCry1 mRNA (Fig 1B), suggesting that the increase in mCRY1 protein under hnRNP Q silencing was not due to altered mRNA stability. Since the translational control of mRNAs is usually mediated by the 5′UTR, we evaluated the association between hnRNP Q and the mCry1 5′UTR. Although hnRNP Q showed affinity to the beads to some degree, this protein strongly bound to the mCry1 5′UTR as expected (Fig 1C). To confirm whether hnRNP Q inhibits the translation of mCry1 via the 5′UTR, we utilized a luciferase reporter system. The 5′UTR of mCry1was inserted at the upstream of firefly luciferase (Fluc) coding sequences, and we conducted luciferase assays 24 hours after transfection into control or hnRNP Q-reduced NIH3T3 cells. Consistent with the result in Fig 1A, the downregulation of hnRNP Q augmented the Fluc level ~40%, compared to control (Fig 1D). Transfection efficiency was normalized with β-gal. Knockdown of hnRNP Q was also confirmed by immunoblotting (Fig 1E). Taken together, these results suggest that hnRNP Q reduced the mCRY1 protein level by suppressing mCry1 translation via the 5′UTR.

### hnRNP Q mainly binds to the forepart of the mCry1 5′UTR

**Fig 2:**
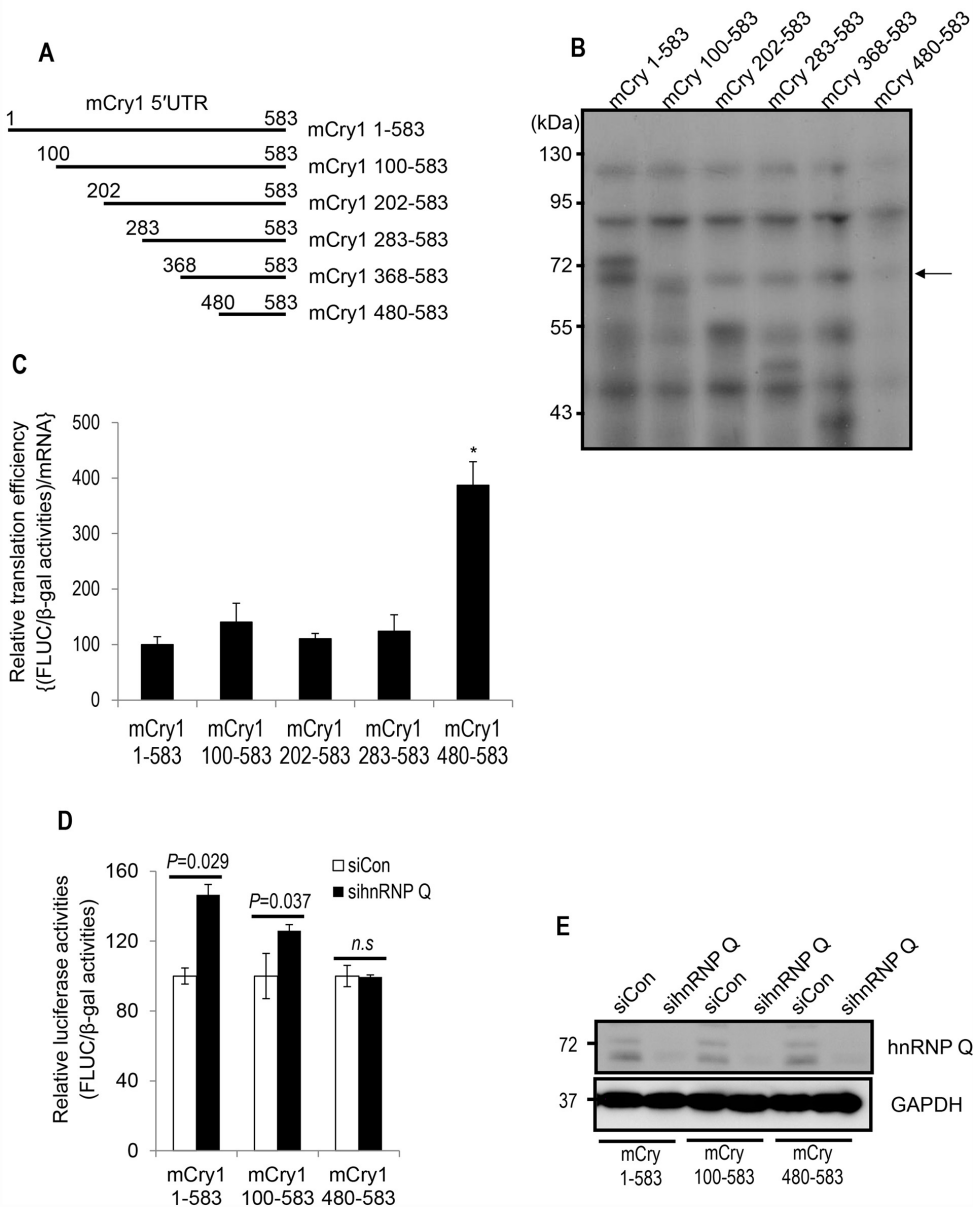
The cis-acting region for hnRNP Q resides in the forepart of the mCry1 5′UTR. (A) Schematic description of serially deleted mCry1 5′UTRs. (B) Cellular proteins that bound to the full-length or truncated forms of the mCry1 5′UTR were analyzed by *in vitro* binding followed by UV-crosslinking assays. The arrow indicates the bands corresponding to hnRNP Q. (C) The translation efficiency of the full-length or deleted forms of the mCry1 5′UTR was determined. Transfection consistency was compensated with β-Gal activity, and translation efficiency was further calculated with mRNA amount derived from each construct. Error bars represent the SEM of four independent experiments. P-value of mCry1 1–583 vs mCry1 480–583 = 0.032. P-value of mCry1 100–583 vs mCry1 480–583 = 0.048. P-value of mCry1 202–583 vs mCry1 480–583 = 0.026. P-value of mCry1 283–583 vs mCry1 480–583 = 0.036. (D) The enhancement of translation mediated by the full-length or deleted forms of the mCry1 5′UTR under hnRNP Q silencing is shown. Error bars represent the SEM of three independent experiments. (E) Downregulation of hnRNP Q was confirmed by immunoblotting.

Although both DNA and RNA consist of nucleotides, RNA is much more structured than DNA. This secondary structure is often highly important to the functionality and regulation of RNA. The interaction between RNA-binding protein and mRNA can be dependent on the secondary structure of mRNA or nucleotide sequence, or both. To determine the cis-acting region of hnRNP Q-mediated translational regulation, serial deletion constructs were generated (Fig 2A). Based on the importance of RNA secondary structure, we also analyzed the secondary structures of full-length (mCry1 1–583) or partially deleted mCry1 5′UTRs (S3 Fig). The mfold Web Server (http://unafold.rna.albany.edu/?q=mfold) was utilized to predict the folded mRNA structures. This software calculates structures by optimizing the thermodynamic free energy. Compared to the other constructs, the mCry1 480–583 construct, which had the shortest sequence length, showed the simplest RNA structure.

Next, we analyzed the binding pattern of hnRNP Q to the full-length or partially deleted mCry1 5′UTR. To this end, we conducted *in vitro* binding followed by UV crosslinking. With other proteins, 68-kDa hnRNP Q showed strong binding affinity to the full-length 5′UTR of the mCry1 mRNA. Once we checked the availability of hnRNP Q antibody for immunoprecipitation experiment (S2A Fig), we confirmed that this 68-kDa protein is undoubtedly hnRNP Q through UV crosslinking followed by immunoprecipitation (S2B Fig). When the first 99 nucleotides were deleted (mCry1 100–583), hnRNP Q still interacted with the deletion transcript, although to a lesser extent. While the association between hnRNP Q and mCry1 5′UTR was observed when the first 367 nucleotides were deleted (mCry1 368–583), an additional 112 nucleotides deletion resulted in the complete loss of hnRNP Q binding (mCry1 480–583) (Fig 2B). Although we couldn’t exactly determine whether the interaction between hnRNP Q and the mCry1 5′UTR is sequence-specific or structure-based, or both, these data suggest that hnRNP Q mainly binds to the forepart of the mCry1 5′UTR. Consistent with this result, translation efficiency was upregulated when hnRNP Q-bindable sequences were removed (Fig 2C). To confirm whether hnRNP Q inhibits the translation of mCry1 via the forepart of 5′UTR, we again utilized a luciferase reporter system (Fig 2D). The augment of Fluc level under hnRNP Q silencing was clearly observed in the full-length construct which highly interacted with hnRNP Q. Fluc expression was still enhanced by hnRNP Q reduction in the mCry1 100–583 construct which showed slightly weaker association with hnRNP Q. Interestingly, however, the upregulation of Fluc expression was not observed by hnRNP Q downregulation in the mCry1 480–583 construct which did not interact with hnRNP Q. Knockdowns of hnRNP Q in each experimental condition were also confirmed by immunoblotting (Fig 2E). These results suggest that hnRNP Q inhibits the translation of mCry1 mRNA through interacting with the forepart of the 5′UTR.

### Downregulation of hnRNP Q alters the oscillation profile of mCRY1 protein

**Fig 3:**
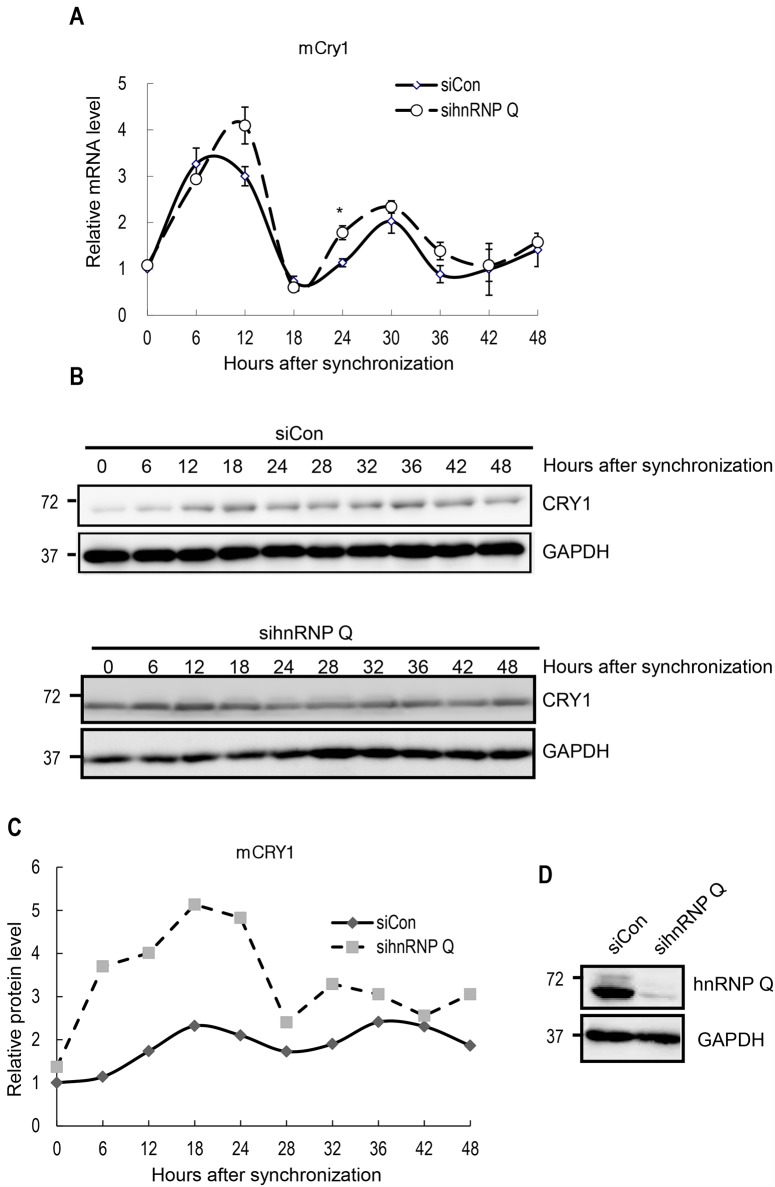
mCry1 protein oscillation is less evident under hnRNP Q silencing. (A) qRT-PCR analysis for endogenous mCry1 mRNA levels after circadian phase synchronization. Error bars represent the SEM of five independent experiments. The initial amount of mCry1 mRNA with siCon was arbitrarily set as 1. *P<0.05. (B) Western blot analysis of mCRY1 in control and hnRNP Q-downregulated cells. (C) The normalized relative expression profile of mCRY1 protein in (B) was plotted. The intensities at 0 hour of siCon group were arbitrarily set as 1. (D) Downregulation of hnRNP Q was confirmed by immunoblotting.

Although Cry genes have important roles in several physiological processes, such as endocrine system functioning, gluconeogenesis, and inflammation, the most well characterized function of the CRY1 protein is circadian timekeeping. To test whether a deficiency in hnRNP Q affects the mRNA and protein oscillation of mCry1, we analyzed the rhythmic profiles of mCry1 mRNA and protein after knockdown of hnRNP Q in cells synchronized by dexamethasone treatment. After temporary treatment with dexamethasone, we harvested NIH3T3 fibroblasts every 6 hours. Using real-time quantitative RT–PCR, we identified that the endogenous mCry1 mRNA profile showed a clear cell-autonomous rhythmicity. This mRNA oscillation seemed almost unchanged under hnRNP Q downregulation (Fig 3A). Interestingly, however, hnRNP Q deficiency resulted in an altered oscillation profile of mCRY1 protein (Fig 3B). The relative band intensities of mCRY1 proteins were analyzed, confirming that the amplitude of the mCRY1 protein oscillation was significantly reduced under hnRNP Q silencing (Fig 3C). Knockdown of hnRNP Q was confirmed by immunoblotting (Fig 3D).

## Discussion

Previously, we already presented the central role of hnRNP Q in molecular circadian rhythm maintenance. In cooperation with hnRNP R and hnRNP L, hnRNP Q destabilizes arylalkylamine N-acetyltransferase (AANAT) mRNA. As a result, the AANAT mRNA oscillating profile showed an increase in peak amplitude and a delay in peak time when three hnRNPs were downregulated [37]. In addition, hnRNP Q rhythmically controls the translation of AANAT mRNA through interacting with IRES (internal ribosome entry site) element within the AANAT 5′UTR. Downregulation of hnRNP Q reduced the peak amplitude of the AANAT protein profile, leading to a deficiency in melatonin production [38]. Furthermore, hnRNP Q has critical roles in the post-transcriptional regulation of core clock genes, including Rev-erb α [30], Per1 [39], and Per3 [40]. In this study, we provide evidences that hnRNP Q controls the translation of mCry1 mRNA and further the oscillation profiles of mCRY1 protein.

In contrast to DNA regulatory sequences, mRNA regulation depends on a combination of the primary (nucleotide sequences) and secondary (stem-loop elements) structures. Although, unfortunately, we could not determine whether the interaction between hnRNP Q and the mCry1 5′UTR is sequence-specific or structure-based, or both, this interaction is critical for translational regulation of mCry1. One important thing is that the translation kinetics of mCry1 mRNA should be controlled in a phase-dependent manner. mCry1 mRNA needs to be efficiently translated into proteins when mCRY1 protein is approaching its peak level. When the mCRY1 protein level is decreasing, the translation of mCry1 mRNA should be less active. This dynamic regulation is possible only if the level or binding affinity of a trans-acting factor is precisely controlled. We already confirmed that the total level of hnRNP Q remains unchanged during circadian oscillation. However, we previously showed that the binding affinity of hnRNP Q to target mRNA is dynamically regulated, raising a possibility that the translation of mCry1 mRNA can be dynamically regulated by hnRNP Q.

It is commonly considered that the nucleotide sequence of UTR regions is less conservative across species than that of protein-coding regions. Interestingly, however, the nucleotide sequence of the Cry1 5′UTR is highly conserved among species (S4 Fig), suggesting that 5′UTR-mediated regulation is critical for Cry1 mRNA fate decision. It remains to be determined whether hnRNP Q-mediated translational regulation of Cry1 mRNA also exists in other species including human. Although we provided some evidence that hnRNP Q is important for mCRY1 protein expression, we cannot exclude the possibility that other RNA-binding proteins are also involved in the translation of mCry1 mRNA. Indeed, as shown in Fig 2B, several unidentified proteins interact with the mCry1 5′UTR, and the binding pattern of those proteins to the mCry1 5′UTR resemble that of hnRNP Q. We further analyzed the binding patterns of other hnRNPs and found that binding pattern of hnRNP I is similar to that of hnRNP Q (S5A Fig). However, reduction of hnRNP I did not affect endogenous level of mCRY1 protein (S5B Fig) and translational efficiency (S5C Fig). Although this result additionally highlights that the interaction between hnRNP Q and mCry1 mRNA is important for translational regulation of mCry1, it would be still necessary to identify other mRNA regulators of core clock genes including Cry1.

Since mCry1 is a main component of the mammalian circadian clock system consisting of the auto-regulatory transcription-translation feedback loop, alteration of the mCRY1 protein oscillation may affect the entire circadian oscillation. Indeed, hnRNP Q deficiency influenced the mRNA profile of mPer3 mRNA and caused the protein fluctuation of mPER1 and mREV-ERB α. It would be of interest to test whether hnRNP Q-mediated regulation is also critical *in vivo* by using transgenic or knockout mice in future studies.

## Methods

### Cell Culture and Dexamethasone Shock

NIH 3T3 cells were obtained from Korean Cell Line Bank (KCLB No. 21658). NIH3T3 fibroblasts were maintained in DMEM (Hyclone) supplemented with 10% fetal bovine serum (Hyclone) and 1% penicillin-streptomycin (Hyclone) in a humidified atmosphere containing 5% CO_2_ at 37°C. To synchronize the circadian rhythms of individual fibroblasts, approximately 2 × 10^5^ cells were seeded in each well of a 12-well plate. When the cells reached semi-confluence, the medium was replaced with medium containing 100nM dexamethasone. After 2hours, this medium was replaced with dexamethasone-free, complete medium (t = 0). Cells were harvested at the indicated times and kept at –70°C for further experiments.

### Plasmid constructions

Standard PCR technique was used to amplify the desired sequences. Amplification of cDNA was performed with Pfu polymerase (Solgent) and confirmed by sequencing. To construct chimeric reporter plasmids, full length and partially-deleted mCry1 5′UTRs were amplified from full-length mouse Cry1 cDNA (accession number NM_007771) by using specific primers. The PCR products were digested with EcoRI and XbaI and then subcloned into the pSK′ vector for *in vitro* binding assays. For luciferase assay, these PCR products were inserted upstream of Fluc coding sequences.

### RNA extraction and cDNA synthesis

Cell pellets were completely dissociated in 500μL of TRI Reagent (Molecular Research Center). Following the addition of 100μL of chloroform, samples were mixed vigorously. Phase separation was conducted by centrifugation. After the aqueous phase was transferred to a new tube, isopropanol-mediated RNA precipitation was performed. Before RNA was dissolved in DEPC-treated water, the RNA pellet was washed with 75% ethanol. RNase-free DNase I (Sigma) was used to remove the contaminating DNA from RNA samples. After inactivation of DNase activity by chelating calcium and magnesium ions with EDTA and by heating, RNA was reverse transcribed using the ImProm-II^™^ Reverse Transcription System (Promega) according to the manufacturer's instructions.

### Quantitative Real-time PCR

Detection and quantification of mRNA was conducted by quantitative RT-PCR (Applied Biosystems). For analysis, cDNA samples were mixed with the FastStart Universal SYBR Green Master Mix (Roche Diagnostics) and primer sets. The relative mRNA level was calculated as values of 2^[C_T_(Rpl32)− C_T_(gene of interest)]. The sequences of the forward and reverse primers are as follows: mouse ribosomal protein L32 (mRpl32), 5′-AACCCAGAGGCATTGACAAC-3′ and 5′-CACCTCCAGCTCCTTGACAT-3′; mouse TATA-binding protein (mTBP), 5′-CAGCCTTCCACCTTATGCTC-3′ and 5′-TTGCTGCTGCTGTCTTTGTT-3′; and mCry1, 5′- CCTTGAAAAGCCTGGGAAAT-3′ and 5′- TCCGCTGCGTCTATATCCTC-3′;

### Protein preparation and immunoblot analysis

For immunoblotting, cells were disrupted with complete protein solubilizing buffer containing 1% SDS and 2M urea in PBS, followed by sonication. Immunoblot analyses were performed with polyclonal anti-CRY1, monoclonal anti-GAPDH (Millipore), polyclonal anti-14-3-3ζ (Santa Cruz Biotechnology), and polyclonal anti-hnRNP Q (Sigma-Aldrich) antibodies. Horseradish peroxidase-conjugated mouse (Thermo Scientific) and rabbit (Jackson ImmunoResearch Laboratories) secondary antibodies were visualized with SUPEX ECL reagent (Neuronex) and a LAS-4000 system (FUJI FILM), according to the manufacturer’s instructions. Acquired images were further analyzed with the Image Gauge program (FUJIFILM).

### RNA interference

The sequences of synthesized siRNAs were as follows. siCon: 5'-UUCUCCGAACGUGUCACGUTT-3' (Samchully Pharm.), and sihnRNP Q: 5'-AGACAGUGAUCUCUCUCAUTT-3' (Dharmacon Research). For siRNA transfection into NIH3T3 cells, the Neon^®^ Transfection System (Invitrogen) was used, according to the manufacturer's instructions.

### *In vitro* binding assay (UV crosslinking, RNA affinity purification)

*In vitro* binding assays through UV crosslinking were performed as described previously [41]. In brief, XbaI-linearized pSK'-mCry1 5′UTR constructs were transcribed using T7 RNA polymerase (Promega) in the presence of [α-^32^P] UTP. 20 μg of whole cell extracts or 40 μg of cytosolic extracts were incubated with labeled RNAs at 30°C. After 30 min of incubation, the mixtures were UV-irradiated on ice for 15 min with a CL-1000 UV-crosslinker (UVP). The samples were detected with autoradiography after SDS-PAGE.

For Streptavidin-biotin RNA-affinity purification of mCry1 5′UTR-binding proteins, XbaI-linearized pSK'-mCry1 5′UTR constructs were transcribed using T7 RNA polymerase (Promega) in the presence of biotin-UTP. Cytoplasmic extracts prepared from NIH3T3 cells were incubated with or without biotinylated RNAs and subjected to streptavidin resin adsorption. For the competition assay, 2-fold molar excess of non-biotinylated competitor RNAs were additionally incubated. Resin-bound proteins were analyzed by sodium dodecyl sulfate-polyacrylamide gel electrophoresis (SDS-PAGE) followed by immunoblotting.

### Transient transfection

For plasmid transfection, NIH3T3 cells were seeded in 24-well plates at a density of 1 × 10^5^ cells per well 12 hours before transfection. Transfections of NIH3T3 fibroblasts with 0.5 μg of luciferase-expressing reporters and 0.2 μg of pCMV·SPORT-β-gal plasmids (Invitrogen) were carried out using Metafectene (Biontex) according to the manufacturer's instructions. After incubation for 48 hours, cells were harvested for further experiments.

### Luciferase assay and β-Gal assay

For the reporter assay, NIH3T3 cells transfected with reporter and control plasmids were lysed in passive lysis buffer (Promega) at 48 hour posttransfection. Fluc activities were determined using the Luciferase Reporter Assay System (Promega) according to the manufacturer’s instructions. β-galactosidase activity was determined from the same lysate with the β-galactosidase Enzyme Assay System (Promega). The transfection efficiency was determined by normalization of Fluc activities with control β-galactosidase activity.

### Immunoprecipitation

For immunoprecipitation, 3 μg of a polyclonal antibody against hnRNP Q or control IgG was added to whole cell lysates (for regular immunoprecipitation) or UV cross-linked and RNase-digested lysates (for UV cross-linking followed by immunoprecipitation) diluted in 700 μl of immunoprecipitation buffer (20 mM HEPES [pH 7.4], 125 mM KCl, 0.05% NP-40, 0.5 mM DTT, 0.5 mM PMSF, and 0.5 mM EDTA). After 16 hours, Protein G agarose beads (Amersham bioscience) were added. After a further incubation for 3 hours, precipitates were detected with immunoblotting or autoradiography after SDS–PAGE.

### Statistical analysis

Unpaired two-tailed Student’s t-test was used for experiments comparing two sets of data unless otherwise noted.

## Supporting Information

S1 Fig The list of hnRNPs that were predicted to interact with mCry1 mRNA. RBPmap program (http://rbpmap.technion.ac.il/index.html) was utilized for computational prediction. Colored letters indicate the expected binding sequences of each hnRNPs.
(TIF)

S2 Fig Confirmation of the interaction between mCry1 mRNA and hnRNP Q. (A) Immunoprecipitation was performed with polyclonal anti-hnRNP Q antibody to check its availability. (B) Identification of the interaction between the mCry1 5′UTR and hnRNP Q by UV crosslinking followed by immunoprecipitation.
(TIF)

S3 Fig RNA secondary structures of full-length or partially-deleted mCry1 5′UTRs. mfold Web Server (http://unafold.rna.albany.edu/?q=mfold) was utilized to predict the folded mRNA structures.
(TIF)

S4 Fig Cry1 5′UTR sequence comparison among species. The nucleotide sequence of the Cry1 5′UTR is well conserved among species. Multiple sequence alignment was performed with Multalin web server (http://multalin.toulouse.inra.fr/multalin/).
(TIF)

S5 Fig hnRNP I does not contribute to expression of mCry1 protein. (A) Cellular proteins that bound to the mCry1 5′UTR were analyzed by Streptavidin-biotin-utilized *in vitro* binding assay followed by immunoblotting. (B) Downregulation of hnRNP I was checked by immunoblotting and endogenous mCry1 protein level was analyzed. (C) The effect of hnRNP I reduction on translation efficiency of 5′UTR of mCry1 was analyzed. The 5′UTR of mCry1 was inserted at the upstream of the Fluc coding sequence. Fluc activity was normalized with β-Gal activity. Error bars represent the SEM of four independent experiments.
(TIF)
