# Supplementary figures and images for: HnRNP Q Has a Suppressive Role in the Translation of Mouse Cryptochrome1
Source: PLoS One. 2016 Jul 8;11(7):e0159018. doi: 10.1371/journal.pone.0159018 (PMC4938544; doi:10.1371/journal.pone.0159018)

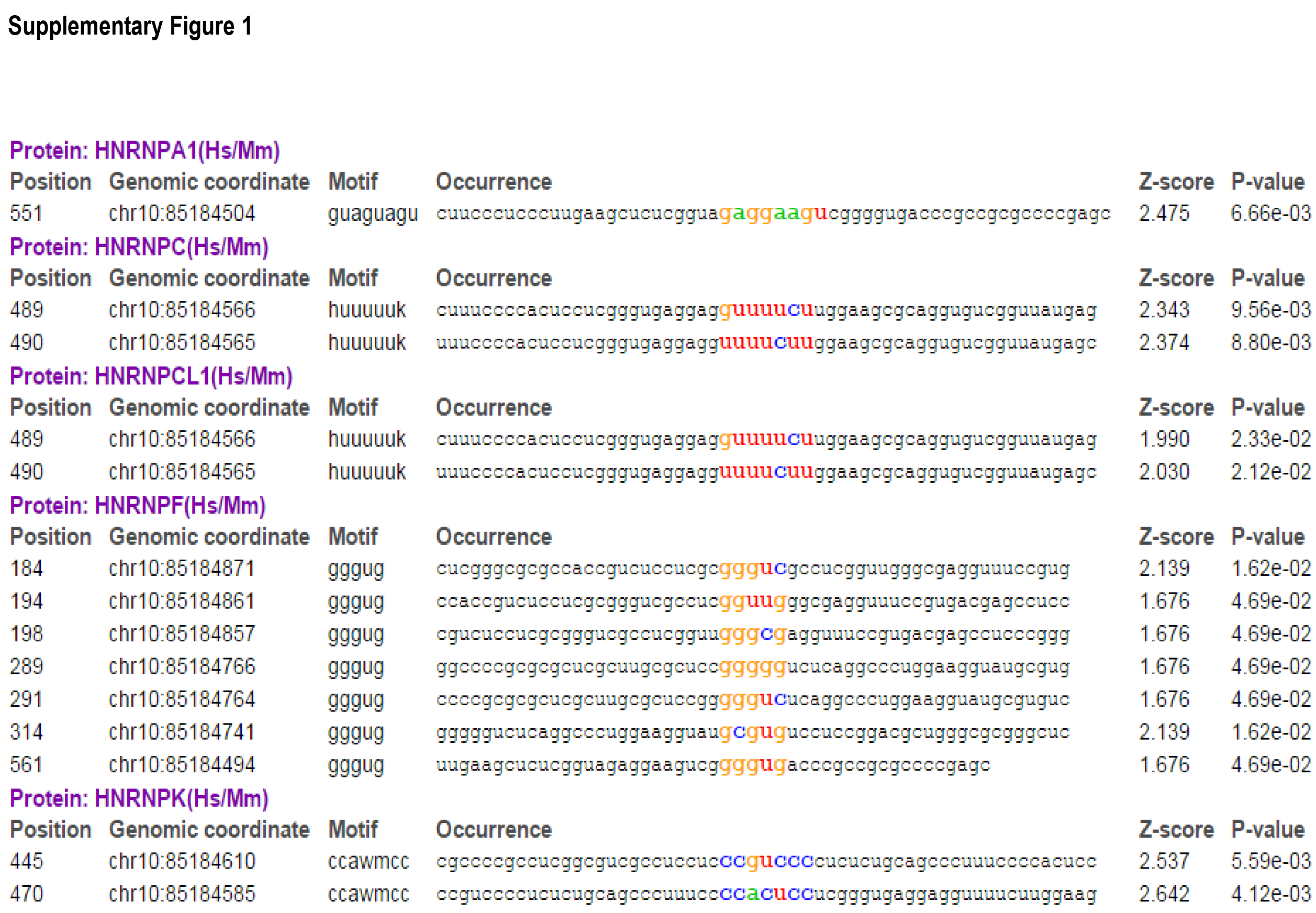

Supplement: S1 Fig — RBPmap program (http://rbpmap.technion.ac.il/index.html) was utilized for computational prediction. Colored letters indicate the expected binding sequences of each hnRNPs. (TIF) [file pone.0159018.s001.tif]

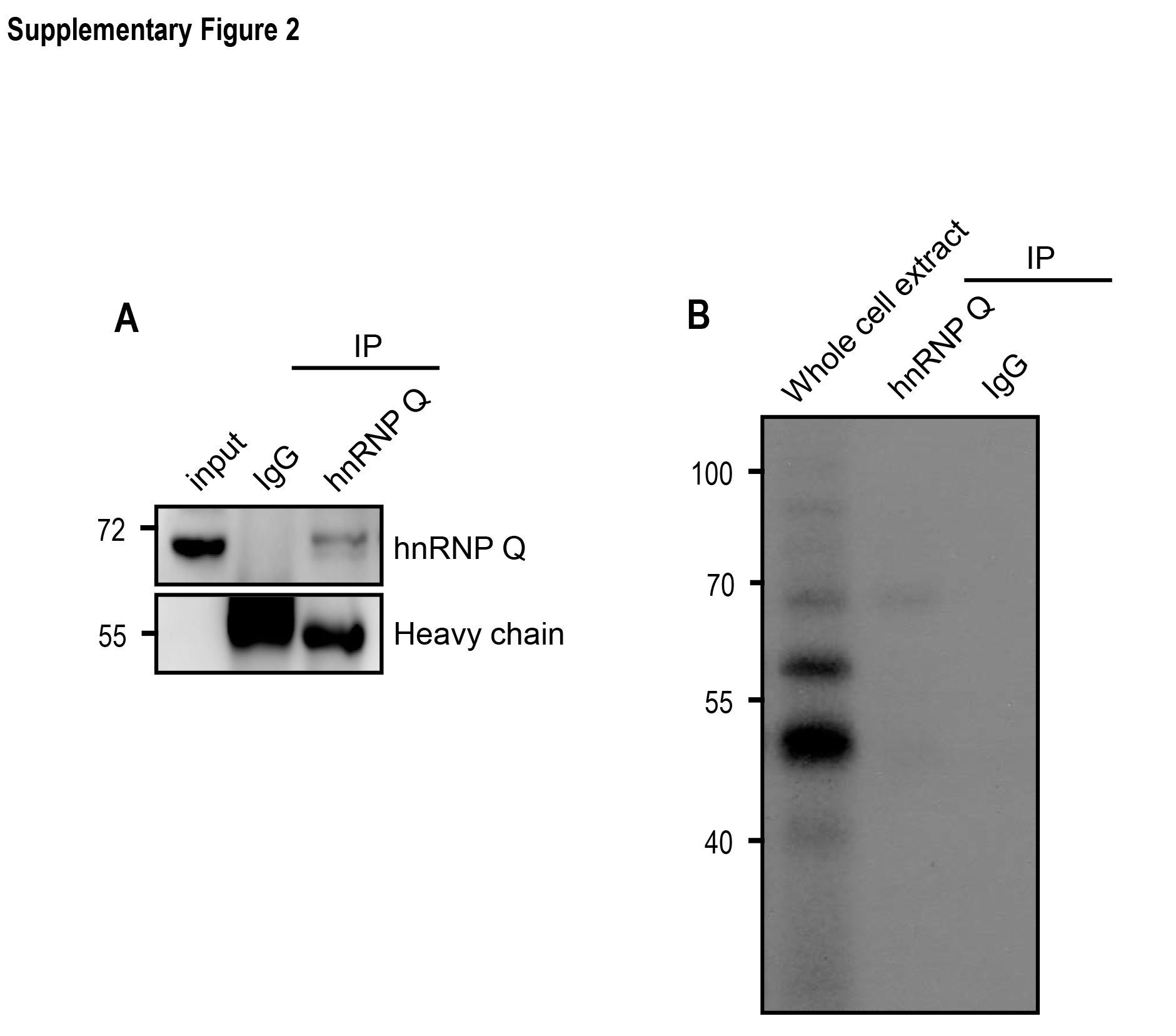

Supplement: S2 Fig — (A) Immunoprecipitation was performed with polyclonal anti-hnRNP Q antibody to check its availability. (B) Identification of the interaction between the mCry1 5′UTR and hnRNP Q by UV crosslinking followed by immunoprecipitation. (TIF) [file pone.0159018.s002.tif]

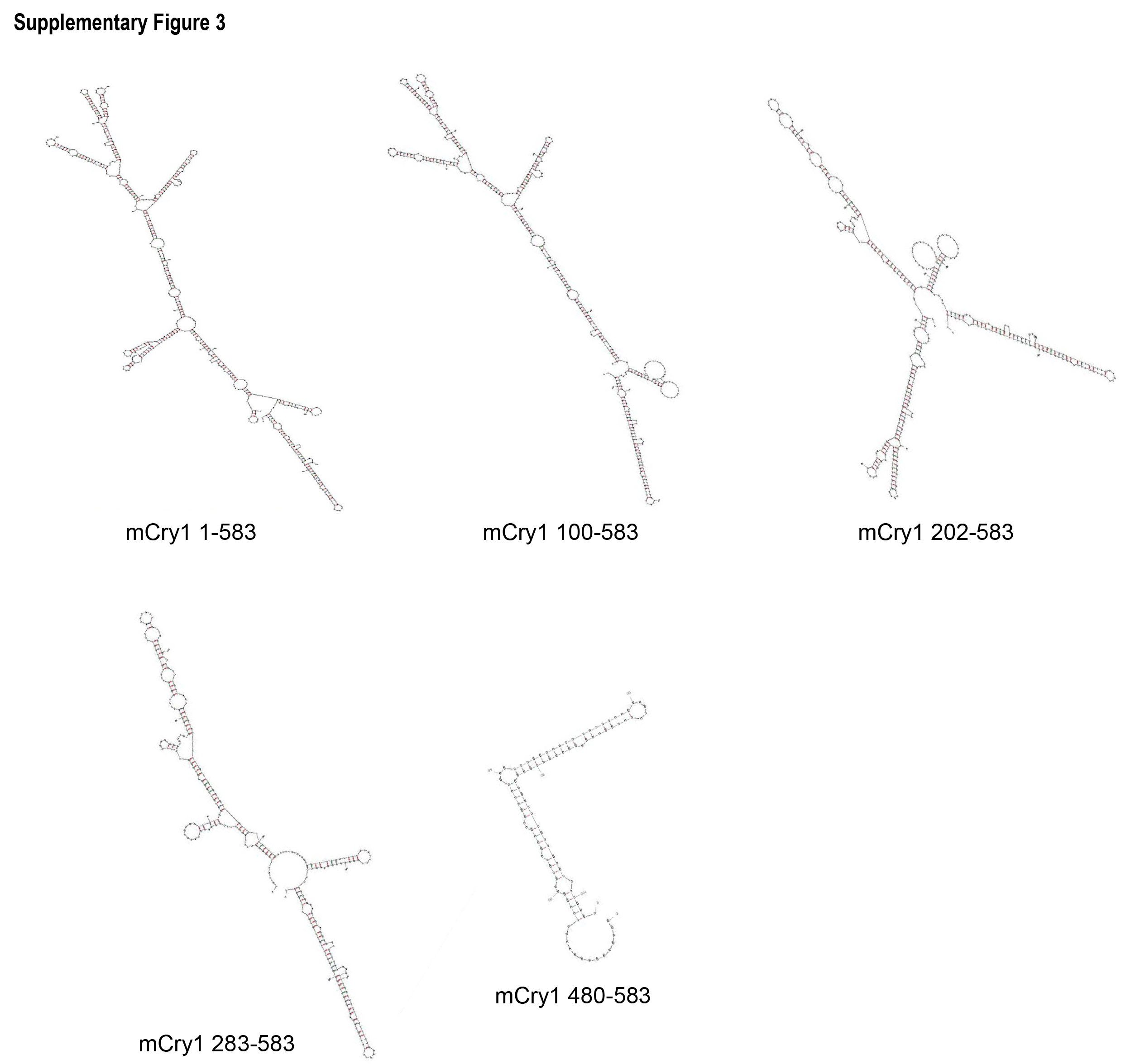

Supplement: S3 Fig — mfold Web Server (http://unafold.rna.albany.edu/?q=mfold) was utilized to predict the folded mRNA structures. (TIF) [file pone.0159018.s003.tif]

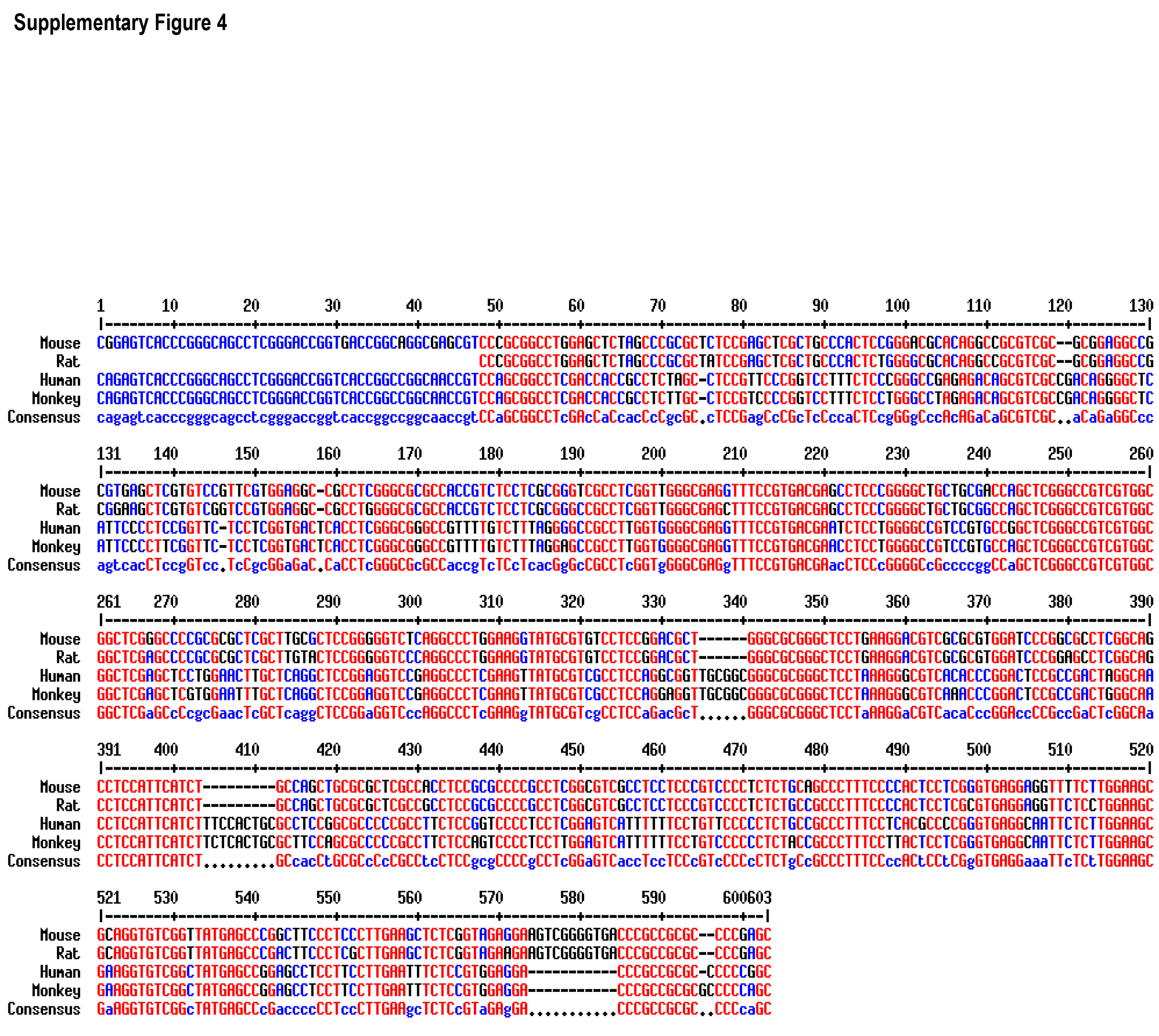

Supplement: S4 Fig — The nucleotide sequence of the Cry1 5′UTR is well conserved among species. Multiple sequence alignment was performed with Multalin web server (http://multalin.toulouse.inra.fr/multalin/). (TIF) [file pone.0159018.s004.tif]

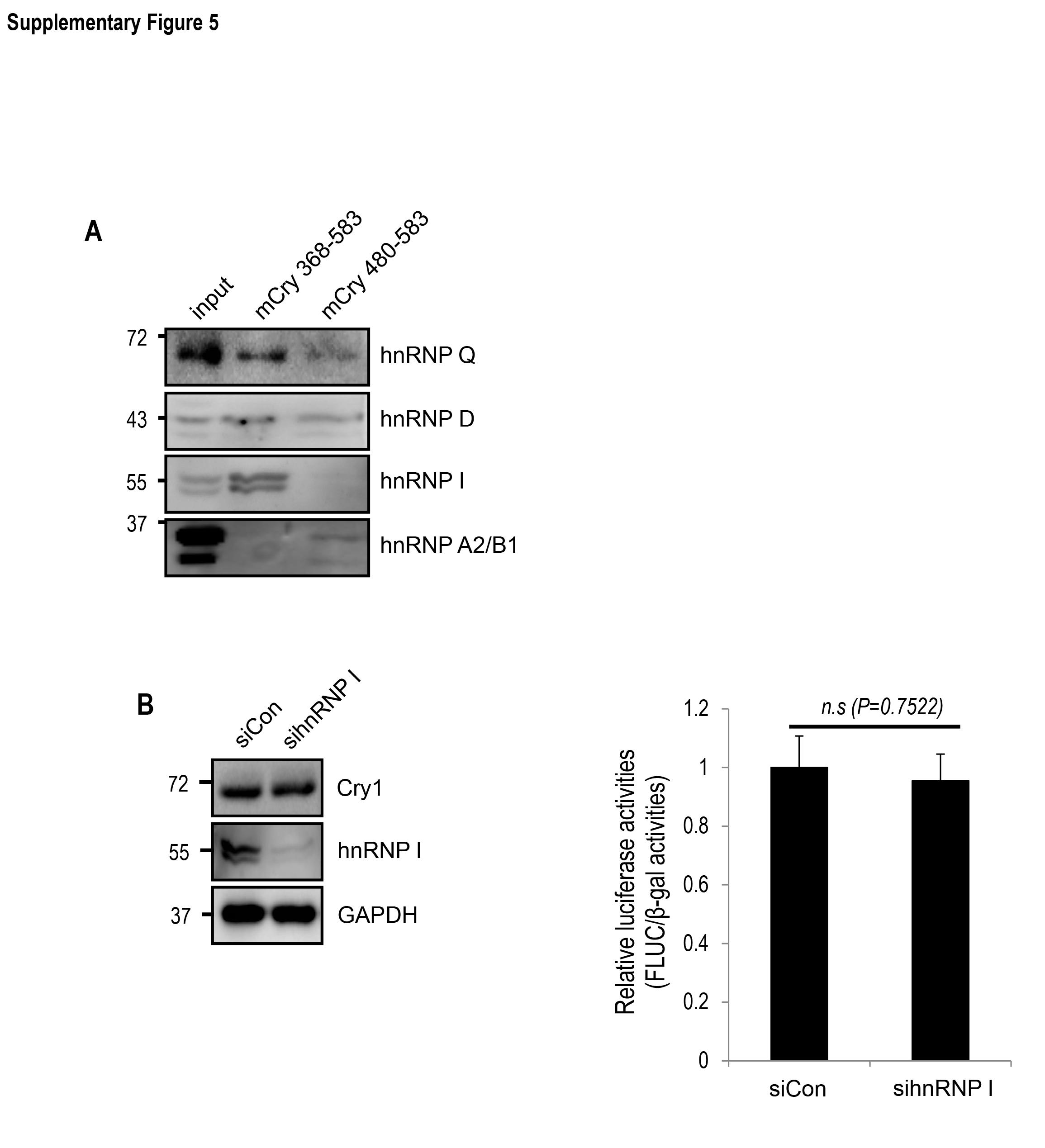

Supplement: S5 Fig — (A) Cellular proteins that bound to the mCry1 5′UTR were analyzed by Streptavidin-biotin-utilized in vitro binding assay followed by immunoblotting. (B) Downregulation of hnRNP I was checked by immunoblotting and endogenous mCry1 protein level was analyzed. (C) The effect of hnRNP I reduction on translation efficiency of 5′UTR of mCry1 was analyzed. The 5′UTR of mCry1 was inserted at the upstream of the Fluc coding sequence. Fluc activity was normalized with β-Gal activity. Error bars represent the SEM of four independent experiments. (TIF) [file pone.0159018.s005.tif]
